# Point-of-Care Ultrasound Use in Cardiac Arrest Patients

**DOI:** 10.3390/diagnostics16101514

**Published:** 2026-05-16

**Authors:** Andrew G. Theophanous, Nina Angeles, Denise Elizondo, Yuriy S. Bronshteyn, Rebecca G. Theophanous

**Affiliations:** 1 College of Medicine, University of Toledo, 3000 Arlington Ave., Toledo, OH 43614, USA; 2 Department of Emergency Medicine, Duke University School of Medicine, 2301 Erwin Rd., Durham, NC 27710, USA; nina.angeles@duke.edu (N.A.); denise.elizondo@duke.edu (D.E.); 3 Durham Veterans Affairs Healthcare System, Durham, NC 27710, USA; yuriy.bronshteyn@duke.edu; 4 Department of Anesthesiology, Duke University School of Medicine, Durham, NC 27710, USA

**Keywords:** ultrasound in cardiac arrest, point-of-care ultrasound, pericardial effusion, cardiac tamponade, aortic dissection, right heart strain

## Abstract

Over 350,000 patients experience out-of-hospital cardiac arrest annually in the United States. Patients presenting to the emergency department (ED) in cardiac arrest are critically ill and require emergent clinical decisions and treatment. Point-of-care ultrasound (POCUS) is useful as a rapid, bedside diagnostic imaging tool to help elucidate possible causes of cardiac arrest and guide management. Using a validated systematic approach such as the SHoC and CASA protocols, clinicians can safely perform POCUS with shortened pauses during pulse checks. POCUS is key in evaluating cardiac arrhythmias such as ventricular fibrillation (VF), which has higher survival rates with defibrillation, versus cardiac standstill, which has higher mortality per recent research studies. When performed by experienced users, POCUS also facilitates identification of reversible causes for improved cardiac arrest patient outcomes and can guide decisions in resuscitative efforts. In summary, this manuscript is a narrative review that illustrates identifiable POCUS findings and synthesizes them to guide potential emergent intervention in patients in cardiac arrest including: ventricular fibrillation, cardiac standstill, pericardial effusion, cardiac tamponade, right heart strain such as from acute pulmonary embolism, reduced cardiac function, wall motion abnormality, ruptured ventricular pseudoaneurysm, and aortic dissection.

**Figure 1:**
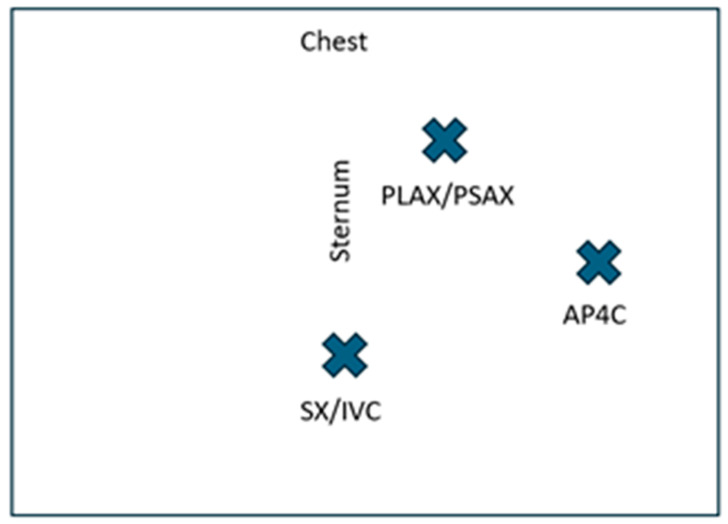
Over 350,000 patients experience out-of-hospital cardiac arrest annually in the United States [1,2]. Patients in cardiac arrest require emergent intervention and treatment. Point-of-care ultrasound (POCUS) has been integrated into resuscitation guidelines to expedite diagnosis and guide management in critically ill patients [3]. Several protocols have been published to guide clinicians to systematically perform focused cardiac POCUS in a time-sensitive manner to detect actionable pathologies [4,5,6,7]. For cardiac arrest patients, POCUS can first determine the presence or absence of cardiac activity. In the largest trial to date, the Reason trial, Gaspari et al. enrolled 793 patients in pulseless electrical activity (PEA) and asystole. They found that 263 patients (33%) had cardiac activity on initial ultrasound, and return of spontaneous circulation (ROSC) was achieved in >50% of these patients. Cardiac activity was associated with increased survival to hospital admission (Odds Ratio 3.6, 2.2–5.9) and to hospital discharge (OR 5.7, 1.5–21.9), whereas lack of cardiac activity was associated with poor survival to hospital discharge (only 0.6% of patients without cardiac activity survived to hospital discharge (95% Confidence Interval 0.3–2.3)) [2]. Additional studies have shown a mortality rate of almost 100% if cardiac standstill was identified during a pulse check. For example, Blaivas et al. described a 100% positive predictive value for mortality in 136 emergency department patients identified with cardiac standstill on POCUS [3,8,9] (Supplementary Video S1). With the appropriate clinical data and context, POCUS can be incorporated into pre-hospital or in-hospital decisions to guide or terminate resuscitative efforts, particularly in patients with asystole. Thus, it is important for clinicians to be able to recognize different cardiac rhythms and pathologies on POCUS [2,3]. This manuscript is an Interesting Images submission written as a narrative review, which is limited by its descriptive nature. Nevertheless, it describes the role of POCUS in cardiac arrest and highlights important POCUS image pathology that clinicians should recognize. Three emergency ultrasound fellowship-trained faculty on the study team reviewed resuscitative guidelines and national POCUS group recommendations for POCUS use in cardiac arrest [1,4,5,6,7]. Together, the authors discussed and came to a consensus about the most common POCUS image findings and pathological illnesses that should be recognized by readers who perform POCUS during cardiac arrest. These were selected as the images highlighted in the Figures and Videos for this manuscript. To ensure appropriate POCUS use during cardiac arrest, Figure 1 shows proper probe positioning for standard cardiac views. POCUS can help distinguish between organized cardiac activity (coordinated contractions of the myocardium resulting in a change in volume of the left ventricle) or disorganized activity (agonal twitching) [10]. For patients with pulseless electrical activity (PEA), those with organized cardiac activity treated with standard advanced cardiac life support (ACLS) interventions demonstrated improved survival to hospital admission compared to those with disorganized activity, 37.7% (95%CI 24.8–50.2%) versus 17.9% (95%CI 10.9–28%). Thus, POCUS may identify subsets of patients with PEA and asystole who may respond differently to resuscitation [10]. Furthermore, POCUS can be used to detect occult ventricular fibrillation (VF). A recent multi-center study of 811 patients by Gaspari et al. showed that cardiac POCUS detected occult VF in 5.3% of patients, which could benefit from defibrillation and potentially change patient outcomes [11] (Supplementary Video S2).

**Figure 2:**
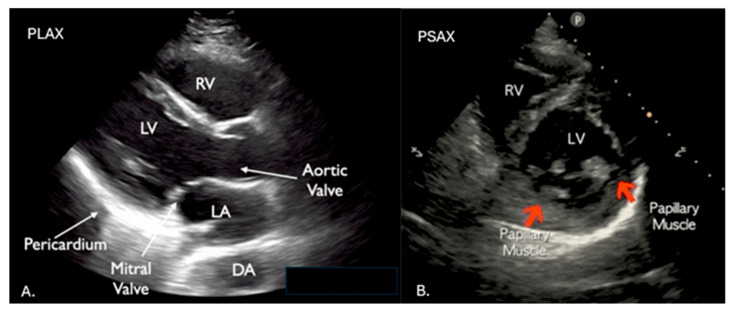

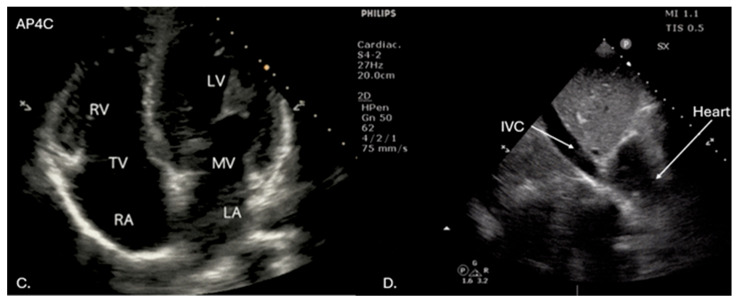
POCUS plays a major role in identifying reversible causes of cardiac arrest. Standard cardiac POCUS views are shown in (**A**–**D**) and include the parasternal long and short axis views (PLAX, PSAX), apical four chamber view (AP4C), subxyphoid view (SX), and inferior vena cava (IVC). Patients may be in shock, which is classified by its cause and includes cardiogenic, obstructive, and hypovolemic. The Sonography in Hypotension and Cardiac Arrest (SHoC) protocol is one example of a systematic process to evaluate potential causes of hypotension and arrest [4,5]. Using this protocol, POCUS users should evaluate the heart with SX and PLAX views, the IVC for increased diameter > 2 cm and reduced respiratory variation (although this is not reliable if the patient is on positive pressure ventilation), anterior and lateral chest views for edema or effusions, the aorta for aneurysm or dissection, the deep veins for thrombus, and the abdomen/pelvis for hemorrhage [4,5]. Challenges of using POCUS during cardiac arrest include its operator dependence, potential for misinterpretation (e.g., distinguishing true cardiac standstill from minimal activity), and the risk of prolonging pulse checks. The Cardiac Arrest Sonographic Assessment (CASA) protocol is an example of a systematic approach to POCUS in cardiac arrest that can help mitigate these challenges [6,7]. Clattenburg et al. found that protocol implementation in 267 cardiac arrest patients over 19 months significantly reduced the duration of cardiopulmonary resuscitation (CPR) interruptions while ultrasound was being performed. Using the CASA protocol, pulse checks with POCUS were 4.0 s shorter (95% CI 1.7–6.3 post-intervention), and POCUS use increased from 64% pre- to 80% post-intervention [6,7]. Additional recommendations for safely incorporating POCUS into cardiac arrest algorithms include having a dedicated person whose only role is to perform the ultrasound and selecting the most experienced POCUS operator to perform the ultrasound. Finally, positioning the ultrasound probe on the chest in preparation for the pulse check and recording the video cine loop then stepping away from the patient for video review can also mitigate pulse check interruptions [6,7].

**Figure 3:**
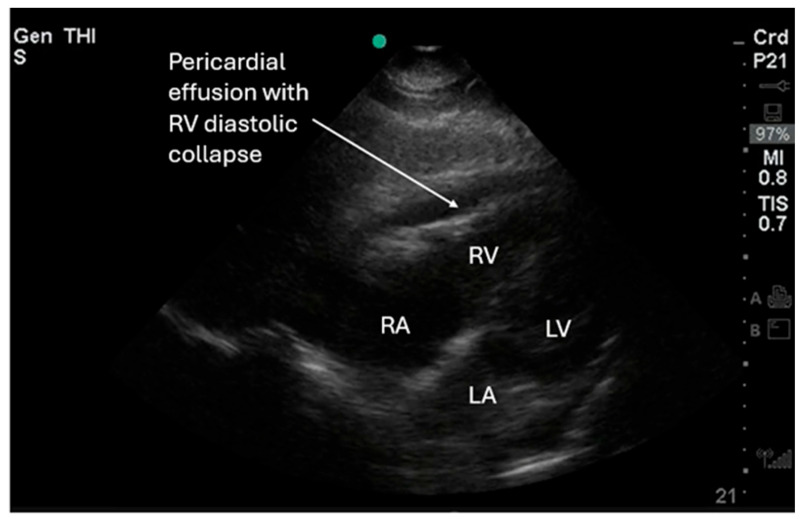
Performing POCUS in cardiac arrest can identify potential causes of contributing factors of the arrest for emergent intervention. Figure 3 shows an SX view of cardiac tamponade with paradoxical right ventricular (RV) diastolic collapse and right atrial (RA) systolic collapse. This may require a bolus of intravenous fluids or emergent pericardiocentesis. In the Reason trial by Gaspari et al., 34 of 793 patients (4.3%) were found to have a pericardial effusion, with 13 requiring pericardiocentesis. This small group had a significantly higher survival to hospital discharge than the rest of the cohort [2]. Pericardial effusions can be small (<2 cm), moderate (2–5 cm), or large (>5 cm). When a pericardial effusion is seen, POCUS users should evaluate for cardiac tamponade physiology (e.g., pericardial effusion with RA early systolic collapse and RV diastolic collapse) [12,13]. Figure 3 and Supplementary Video S3 show cardiac tamponade in PLAX and SX views. Additional views, such as the IVC, should be used in cases of suspected tamponade for verification [14,15,16]. Studies show that a dilated IVC > 2 cm with <50% respiratory variation is suggestive of a volume overload state, whereas a normal IVC is <2 cm with >50% respiratory variation [12,13,16]. The IVC diameter should be measured about 2 cm distal to the hepatic vein confluence away from the cavo-atrial junction. POCUS users should note that IVC assessment is not reliable in patients with positive pressure ventilation, right heart elevated pressures, pulmonary hypertension, tricuspid regurgitation, or increased abdominal pressure [14,15,16].

**Figure 4:**
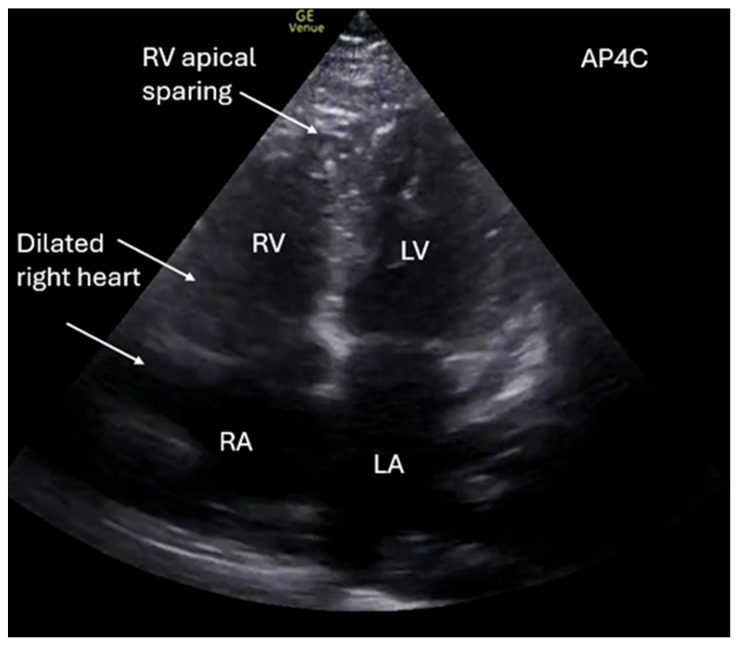
Right heart strain can be acute or chronic, appearing with a dilated right atrium (RA) and right ventricle (RV) as in Figure 4 (AP4C view). The most common cause of acute right heart strain is pulmonary embolism. In contrast, chronic right heart failure can be caused by pulmonary hypertension, chronic lung disease, obstruction in outflow, left-sided heart failure, and valvular disease [14,17]. There are multiple signs that indicate right heart strain on POCUS, and the literature is mixed on which of these is the best indicator for acute right heart strain. More easily recognized POCUS findings are a dilated right atrium/ventricle on AP4C view (Figure 4 and Supplementary Video 4) or a D-sign present on PSAX view, which is a flattening of the septum with bowing into the RV [12,13]. Additional findings in acute right heart strain with high sensitivity and specificity are tricuspid annular plane systolic excursion (TAPSE) < 17 mm, which is measuring how far the tricuspid valve annulus is moving, and McConnell’s sign, which is characterized by right ventricular immobility with hyperkinesis of the apical tip on A4C view (Figure 4) [15,16,17].

**Figure 5:**
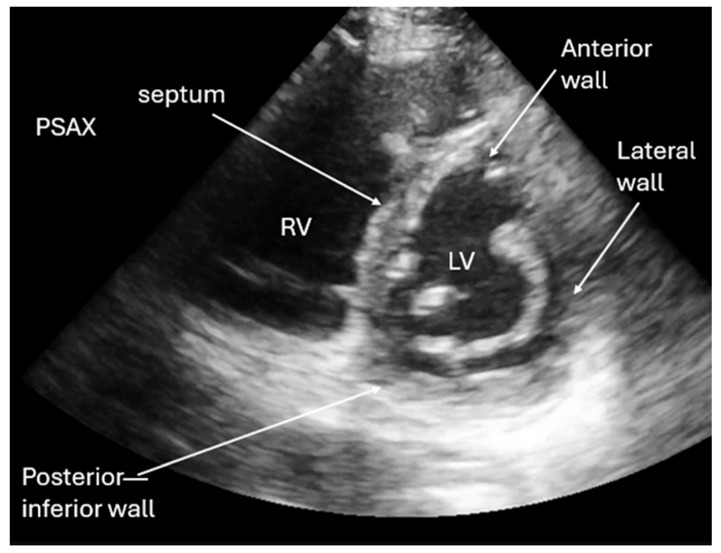
Patients in cardiogenic shock will have a reduced ejection fraction (end point septal separation (EPSS) > 7 mm, which is the measurement between the septum and the tip of the mitral view on M-mode in PLAX view) [12,13,16]. Patients who arrested from an acute myocardial infarction (MI) may have ST segment elevation on their electrocardiogram (ECG). POCUS is important in identifying wall motion abnormalities in PSAX view or AP4C/AP2C views. Figure 5 demonstrates the regions in PSAX view for detecting wall motion abnormality in a patient with acute MI. By localizing reduced cardiac function to one area of coronary vessel blood supply, patients can undergo expedited catheter-directed therapy via cardiac stent or balloon angiography. Reduced door-to-balloon times under 90 min demonstrates improved patient outcomes [18,19]. For example, the left anterior descending (LAD) artery supplies blood flow to the anterior heart and septum. The left circumflex artery supplies blood to the lateral heart. The right coronary artery or posterior descending artery (PDA) usually supplies the posterior or inferior portion of the heart [16,18]. Figure 5 and Supplementary Video S5 show an example of anterior/septal wall motion abnormality in a patient with an LAD occlusive MI.

**Figure 6:**
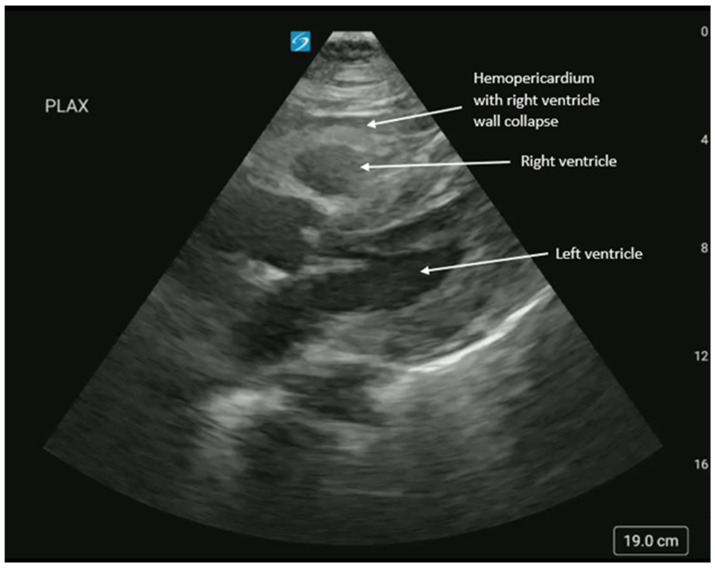
A ventricular aneurysm or pseudoaneurysm can rupture and cause a pericardial effusion with obstruction of cardiac outflow. Although rare, rupture has high morbidity and mortality rates [20,21,22]. A pseudoaneurysm is the separation of cardiac muscle layers between the myocardium and epicardium and is caused by post-cardiac-surgery trauma, infection, or myocardial infarction. A pseudoaneurysm differs from a true aneurysm in that myocardial tissue is absent in the wall, and it forms as a narrow neck between the ventricle and paraventricular chamber instead of a broad-based ventricular outpouching. A blood vessel or the myocardial wall ruptures but is contained within the pericardium, by thrombus, or adhesions [20,21,22]. Figure 6 and Supplementary Video S6 show a ruptured right ventricular (RV) pseudoaneurysm with coagulating blood in the pericardial space in a patient with cardiac arrest. This was suspected to be caused by traumatic rib fractures during CPR, but ventricular pseudoaneurysm could also occur spontaneously [20,21,22]. When performing POCUS, ED physicians should be careful to distinguish between epicardial fat, which is located anterior to the heart and is hyperechoic, compared to fluid, which is anechoic when acute or heterogeneous when subacute (coagulated) [14,16]. Physicians may identify pericardial blood or paradoxical right ventricular collapse indicating cardiac tamponade. Ventricular pseudoaneurysm management can be conservative, involve percutaneous closure using cardiac angiography, or open surgical repair [20,23].

**Figure 7:**
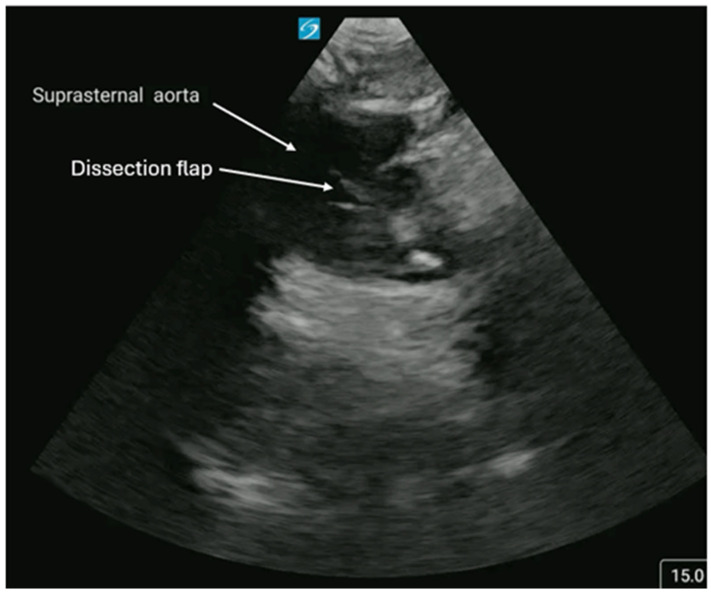
Aortic pathology can also be detected in cardiac views. The aortic arch must be viewed in the suprasternal notch (SS) view by placing the probe superior to the sternum with the marker pointing towards the patient’s head and then rotated clockwise to the one o’clock position. Figure 7 and Supplementary Video S7 are examples of an SS view that visualizes a dissection flap in the aortic arch. PLAX and AP4C views visualize parts of the descending aorta and can also detect a dissection flap. PLAX view also shows the aortic root, which if measuring > 4 cm in diameter is defined as an aneurysm [14,15,16]. Supplemental Table S1 summarizes the major diagnostic findings for each cardiac arrest pathology described and the recommended interventions. In summary, POCUS is a validated, guideline-endorsed tool integrated into the resuscitation of cardiac arrest patients. Its integration into clinical pathways for cardiac arrest can expedite diagnosis and facilitate critical time-sensitive bedside interventions for improved patient care. We acknowledge that this manuscript is limited by its primarily descriptive nature and largely summarizes well-established concepts. Clinicians should be careful when performing POCUS so that it does not hinder chest compressions or extend pulse checks beyond the ACLS guidelines’ recommended 10 s pause. Multiple studies have shown controversy in performing POCUS during cardiac arrest due to concerns for prolonged pulse checks and poorer patient outcomes [4,5,6,7,13,14,15]. However, the consensus based on highly quality studies and systematic reviews is to (1) have POCUS performed by a trained POCUS expert, (2) have the probe ready on the chest prior to cessation of chest compressions, and 3) save a 6 s video cine clip during the pulse check so that the probe can be immediately moved to resume compressions [4,5,6,7,13,14,15]. Future prospective studies and systematic reviews should further define the role of POCUS in cardiac arrest patients and its impact on patient care.

## Data Availability

The original contributions presented in this study are included in the article. Further inquiries can be directed to the corresponding author.

## Supplementary Materials

The following supporting information can be downloaded at https://www.mdpi.com/article/10.3390/diagnostics16101514/s1, Video S1: SX view of cardiac standstill; Video S2: SX view of ventricular fibrillation; Video S3: SX view of pericardial effusion and cardiac tamponade with diastolic right ventricular collapse; Video S4: AP4C view with right atrial/ventricular dilation and apical sparing in the setting of acute pulmonary embolism; Video S5: PSAX view of reduced cardiac ejection fraction with anterior wall motion abnormality in a patient with LAD myocardial infarction; Video S6: PLAX view of right ventricular pseudoaneurysm rupture with coagulated pericardial blood and right ventricular wall collapse; Video S7: Suprasternal notch view of aortic arch dissection flap. Table S1: Major diagnostic findings for each cardiac arrest pathology described, recommended locations for POCUS scanning, and intervention.

## Abbreviations

The following abbreviations are used in this manuscript:

POCUS	Point-of-care ultrasound
VF	Ventricular fibrillation
ED	Emergency department
PEA	Pulseless electrical activity
RV	Right ventricle
LV	Left ventricle
PE	Pulmonary embolism
ROSC	Return of spontaneous circulation
CPR	Cardiopulmonary resuscitation
