# Supplementary material for: Point-of-Care Ultrasound Use in Cardiac Arrest Patients
Source: Diagnostics (Basel). 2026 May 16;16(10):1514. doi: 10.3390/diagnostics16101514 (PMC13205991; doi:10.3390/diagnostics16101514)
Supplement: Supplementary file 1 [file diagnostics-16-01514-s001.zip › S8 Supplemental Table 1 Cardiac Arrest.pdf]

S8 Supplemental Table S1. Major diagnostic findings for each cardiac arrest pathology described, recommended locations for POCUS scanning, and intervention

Cardiac arrest pathology	Diagnostic findings on ultrasound	Optimal views for detection	Treatment
Cardiac tamponade	Pericardial effusion with paradoxical RV diastolic collapse and RA systolic collapse. Dilated IVC.	SX or PLAX, IVC views	Intravenous fluid bolus or pericardiocentesis
Acute right heart strain	Dilated RV with septal flattening away from the LV (D-sign); Dilated RA and RV with apical sparing (McConnell's sign). Dilated IVC. TAPSE <17mm.	PSAX; AP4C, IVC views	Fluid bolus, intravenous pressors, systemic or interventional thrombolysis or central pulmonary clot retrieval
Cardiogenic shock with acute myocardial infarction	Reduced ejection fraction; wall motion abnormality in affected coronary vessel distribution	All views; PSAX or AP2C	Aspirin, systemic anticoagulation, and cardiac catheterization lab for coronary stent placement or balloon angioplasty. Cardiac vessel bypass surgery for multivessel disease.
Ventricular aneurysm or pseudoaneurysm rupture	Anechoic or hypoechoic blood in pericardium with RV diastolic collapse	SX or PLAX	Conservative, percutaneous closure using cardiac angiography, or open surgical repair
Aortic dissection	Dissection flap within the ascending or descending aorta or arch	PLAX, PSAX, AP4C, Suprasternal notch view	Surgical repair for type A ascending aorta dissection and blood pressure/heart rate reduction for type B descending aorta

Key: RA Right atrium, RV (right ventricle), IVC (inferior vena cava), SX (subxiphoid), PLAX (parasternal long axis), PSAX (parasternal short axis), AP4C (apical four chamber), AP4C (apical two chamber), TAPSE (tricuspid annular plane systolic excursion)
